# DNMBP-AS1 Regulates NHLRC3 Expression by Sponging miR-93-5p/17-5p to Inhibit Colon Cancer Progression

**DOI:** 10.3389/fonc.2022.765163

**Published:** 2022-04-27

**Authors:** Lijie Yang, Tiecheng Yang, Huaqiao Wang, Tingting Dou, Xiaochang Fang, Liwen Shi, Xuanfei Li, Maohui Feng

**Affiliations:** Department of Gastrointestinal Surgery, Zhongnan Hospital of Wuhan University, Clinical Medical Research Center of Peritoneal Cancer of Wuhan, Clinical Cancer Study Center of Hubei Provence, Key Laboratory of Tumor Biological Behavior of Hubei Provence, Wuhan, China

**Keywords:** colon cancer, DNMBP-AS1, miR-93-5p, miR-17-5p, NHLRC3, ceRNA

## Abstract

Long non-coding RNAs (LncRNAs) act as competing endogenous RNAs (ceRNAs) in colon cancer (CC) progression, *via* binding microRNAs (miRNAs) to regulate the expression of corresponding messenger RNAs (mRNAs). This article aims to explore the detailed molecular mechanism of ceRNA in CC. Top mad 5000 lncRNAs and top mad 5000 mRNAs were used to perform weighted gene co-expression network analysis (WGCNA), and key modules were selected. We used 405 lncRNAs in the red module and 145 mRNAs in the purple module to build the original ceRNA network by online databases. The original ceRNA network included 50 target lncRNAs, 41 target miRNAs, and 34 target mRNAs. Fifty target lncRNAs were used to establish a prognostic risk model by univariate and least absolute shrinkage and selection operator (LASSO) Cox regression analyses. LncRNAs in the risk model were used to build the secondary ceRNA network, which contained 9 lncRNAs in the risk model, 35 miRNAs, and 29 mRNAs. Survival analyses of 29 mRNAs in the secondary ceRNA network have shown HOXA10 and NHLRC3 were identified as crucial prognostic factors. Finally, we constructed the last ceRNA network including 5 lncRNAs in the risk model, 8 miRNAs, and 2 mRNAs related to prognosis. Quantitative real-time polymerase chain reaction (qRT-PCR) results revealed that DNMBP-AS1 and FAM87A were down-regulated in CC cells and tissues. Function assays showed that over-expression of DNMBP-AS1 and FAM87A inhibited CC cells proliferation and migration. Mechanism study showed that DNMBP-AS1 served as miR-93-5p/17-5p sponges and relieved the suppression effect of miR-93-5p/17-5p on their target NHLRC3. Our study suggested that DNMBP-AS1 inhibited the progression of colon cancer through the miR-93-5p/17-5p/NHLRC3 axis, which could be potential therapeutic targets for CC.

## Introduction

According to Global Cancer Statistics 2020, colon cancer (CC) ranks third in terms of incidence but second in terms of mortality. Parts of Europe, Australia/New Zealand, Northern America, and Eastern Asia have the highest incidence of colon cancer (1). Furthermore, incidence rates of colon cancer have been still steadily rising in many countries (1). Colon adenocarcinoma (COAD) accounts for more than 90% of colon cancer (2). COAD is prone to metastases, leading to a poor prognosis for colon cancer patients despite improved diagnosis and treatment techniques (3, 4). Therefore, it is critical to further explore the molecular mechanism of CC and find new therapeutic targets to improve prognosis.

Long non-coding RNAs (LncRNAs) are identified as a group of RNAs without protein-coding ability and larger than 200 nucleotides to distinguish them from small non-coding RNAs (5). LncRNA plays a key role in gene regulation, as they can affect cellular proliferation, migration, and genomic stability (6). In recent years, transcriptome sequencing has revealed that thousands of lncRNAs with aberrant expression are related to different cancers (6). Mechanisms of lncRNAs function in carcinogenesis include enhancing the chromatin state and methylation, maintaining the stability of proteins or protein complexes, and acting as a sponge for microRNA (miRNA) inhibition (7). Some lncRNAs have already been linked to poor prognosis in multiple tumors (8). A previous study has shown that linc-UBC1 over-expression is associated with poor overall survival (OS) and advanced tumor stage in various cancers (9). The high expression level of lncRNA SNHG6 is correlated with tumor invasion and advanced Tumor-Node-Metastasis (TNM) stage in human cancers (10).

As mentioned above, lncRNAs act as competing endogenous RNAs (ceRNAs) in many kinds of cancers, by sponging miRNAs and reducing inhibition of their target messenger RNAs (mRNAs) (8). A recent study has shown that lncRNA DNAJC3-AS1 may promote colon cancer progression *via* regulating the miR-214-3p/LIVIN axis (11). LncRNA MINCR could up-regulate CTNNB1 expression by sponging miR-708-5p to promoting colon cancer cell proliferation and migration (12).

In this study, we used weighted gene co-expression network analysis (WGCNA) to identify the lncRNAs and mRNAs most associated with COAD clinical traits. We constructed the prognostic risk model based on lncRNAs and performed survival analysis of related mRNAs. Thus, the ceRNA network associated with survival in COAD was established. Finally, we further studied the functions and mechanisms of DNMBP-AS1 and FAM87A in colon cancer to find effective therapeutic targets for CC patients.

## Materials and Methods

### Data Pre‐Processing

We downloaded RNA-seq data including lncRNAs and mRNAs expression data and clinical information of colon cancer patients from The Cancer Genome Atlas (TCGA) dataset (https://cancergenome.nih.gov/). In this study, inclusion criteria were as follows: (1) patients with a diagnosis of COAD; (2) patients with complete clinical information on age, gender, vital status, pathologic T stage, pathologic N stage, pathologic M stage, and tumor stage; (3) patients with specific follow‐up time. Then, we treated the RNA matrix in the following way: (1) same RNA with more than one row were averaged; (2) RNAs with low value (expression <1) were excluded; (3) edgeR package of R software was used to normalized the RNA-seq data of ‘Level‐3’ read counts.

### WGCNA of lncRNAs and mRNAs

WGCNA is a systems biology method for finding gene modules with highly correlated expression levels and for relating them to clinical traits. Therefore, WGCNA is widely used to identify and screen biomarkers or therapeutic targets (13). Top 5000 mad lncRNAs and mRNAs were selected for further analysis with WGCNA package. First, all samples were clustered to construct a sampleTree and detect outliers based on cut height. Sample dendrogram and trait heatmap were used to develop networks to investigate the relationship between the corresponding sample gene expression data and clinical phenotypes. Then we selected β = 4 for lncRNAs and β = 6 for mRNAs as the soft‐thresholding parameters to construct the adjacency matrix. Next, we transformed the adjacency matrix into the topological overlap matrix. According to the topological overlap matrix (TOM)‐based dissimilarity measure, genes with highly absolute correlations were clustered into the same module to generate a cluster dendrogram (deep-split = 2, minimum cluster size = 30, cut height = 0.25). To visually represent the relationships between modules and clinical features of COAD, we calculated the Pearson correlation coefficient and plotted heatmap. Gene modules were significantly correlated with traits when P-value < 0.05. High correlation modules were selected as key modules for further analysis.

### Construction of ceRNA Network

We constructed the ceRNA network based on lncRNAs and mRNAs in the key modules. The MiRcode (http://www.mircode.org/) database was used to predict lncRNA-miRNA interactions according to lncRNAs in the key module. StarBase (http://starbase.sysu.edu.cn/index.php) database was used to transform candidate miRNAs into human mature miRNA names. TargetScan (http://www.targetscan.org/vert_72/), miRTarBase (http://mirtarbase.mbc.nctu.edu.tw/php/search.php), and miRDB (http://www.mirdb.org/) databases were used to predict miRNA-mRNA interactions, which must exist in three databases simultaneously. Finally, we obtained the overlaps between the candidate mRNAs and mRNAs in the key module. Cytoscape 3.7.1 software was used to construct and visualize the lncRNA‐miRNA ‐mRNA network.

### Functional Annotation Analysis

We used Kyoto Encyclopedia of Genes and Genomes (KEGG) and Gene Ontology (GO) analyses to deeply understand the biological function of target mRNAs. Then, the clusterProfiler package was used to conduct functional enrichment analyses of target mRNAs, and the enrichplot package and ggplot2 package were used to visualize the results of the enrichment analyses. Top enriched terms with P-value <0.05 were selected.

### Construction of lncRNAs-Based Prognostic Risk Model

In order to construct a lncRNA-based risk model to predict OS of COAD patients, the following steps were performed with survival package, glmnet package, survminer package, and time receiver operating characteristic (ROC) package. Firstly, univariate COX regression analysis was used to select lncRNAs from the original ceRNA network. Next, lncRNAs with P-value <0.1 were included in the least absolute shrinkage and selection operator (LASSO) regression analysis. Lambda.min of LASSO analysis was used to construct lncRNA prognostic signature. The following formula was used to calculate the risk score of patients: Risk score = β_RNA1_ * exp_RNA1_ + β_RNA2_ * exp_RNA2_ + … + β_RNAn_ * exp_RNAn_, where β _RNA_ indicated the prognostic coefficient of lncRNA, and exp_RNA_ stood for the expression level of lncRNA. At last, the risk score of every COAD patient was calculated based on the prognostic signature. All patients were divided into high-risk and low-risk sets according to the median risk score value. The Kaplan–Meier curve was used to evaluate OS, and ROC curve and areas under the curve (AUC) were used to evaluate the predictive accuracy of the prognostic risk model. Heatmap of clinical traits and the expression levels of lncRNAs was constructed by pheatmap package.

### Construction of Nomogram

Nomogram was used to demonstrate a risk model based on lncRNAs and clinical characteristics for assessing OS in COAD patients. Univariate and multivariate COX regression analyses were performed for clinical characteristics using survival package. The P-value equal to 0.05 was selected to be the significant threshold. Clinical features with a significant threshold in multivariate analysis were identified as independent prognostic factors and included in nomogram. Finally, rms package was used to establish a nomogram. The C-index, calibration curve, and ROC analysis were used to describe the prediction value of the nomogram.

### Survival Analysis of mRNAs

We integrated mRNA expression and clinical prognostic information (vital status and follow‐up time) of COAD patients. Survival package in R software was used to perform survival analysis. The mRNAs with P-value <0.05 were considered as crucial prognostic factors.

### Tissue Samples

Colon cancer and adjacent normal tissues were collected from patients undergoing radical resection and with a postoperative pathology diagnosis of colon cancer at Zhongnan Hospital of Wuhan University from October 2019 to September 2020. Written informed consent was obtained from each patient before surgery and the patient protocols were approved by the hospital’s ethics committee. The samples were stored at −80°C immediately after resection until use.

### Cell Culture

Colon cancer cell lines (SW480, HCT 116, SW620, HT-29, and DLD-1) and normal human colon mucosal epithelial cell line (NCM460) were purchased from the Cell Bank of the Chinese Academy of Sciences. The cells were cultured in Dulbecco’s modified Eagle’s medium (DMEM; BI, Israel) supplemented with 10% fetal bovine serum (FBS; BI, Israel) at 37°C with 5% CO_2_.

### Total RNA Extraction, Reverse Transcription, and Quantitative Real-Time Polymerase Chain Reaction (qRT-PCR)

The EASYspin RNA Mini Kit (Aidlab Biotechnologies Co., Ltd, China) was used for total RNA extraction according to the manufacturer’s instructions. Reverse transcription was performed using the RevertAid First Strand cDNA Synthesis Kit (Thermo Scientific, USA) with 2 µg total RNA. PCR was conducted using the SYBR Green qPCR Mix (High ROX) (Monad, China) on a CFX96 Touch Real-Time PCR Detection System (Bio-Rad, USA). Glyceraldehyde 3-phosphate dehydrogenase (GAPDH) and U6 were used as internal controls and relative RNA expression was quantified using the 2^-ΔΔCt^ method (14). The primers (Tsingke, China; RiboBio, China) were listed in **Supplementary Table 1**.

### Transfection

To over-expressed DNMBP-AS1 and FAM87A, lentivirus was designed and constructed by the Genechem Company (China). Stable transfected SW480 cells were obtained by selection with puromycin after lentivirus infection according to the manufacturer’s protocol. The miR-93-5p/17-5p mimics and mimics negative control (GenePharma, China) were transfected into SW480 cells using Lipofectamine 3000 (Invitrogen, USA) following the manufacturer’s instructions.

### Cellular Proliferation Assays

In the Cell Counting Kit-8 (CCK-8) assay, stable transfected SW480 cells were seeded into 96-well plates at a concentration of 2x10^3^ cells/well. 10 µL of CCK-8 reagent were added to each well and the cells were incubated for a further 2 h. To estimate cell viability, the absorbance of each well was measured at 450 nm every 24 h for 4 days.

In clone-formation assay, 1x10^3^ cells were plated in each well of a six-well plate. After culturing for 10 days, the cells were fixed with 4% paraformaldehyde for 30 minutes, stained with 0.1% crystal violet for 30 minutes, photographed, and counted the number of clones in each plate.

### Wound-Healing Assay and Transwell Assay

In the wound-healing assay, stable transfected SW480 cells were seeded into six-well plates. After culturing for 24 h, the cells were scratched with a linear wound with a 200 µL pipette tip, washed with phosphate buffered saline (PBS), imaged with an inverted microscope (Nikon, Japan) at 0 and 24 h, and measured the wound width using Image J software (version 1.53).

In the transwell assay, cellular invasion and migration assays were performed using transwell inserts (Corning, USA) coated with or without Matrigel, respectively. Stable transfected SW480 cells suspension (5×10^4^ cells) was added to the upper chamber, and DMEM containing 20% FBS was added to the lower chamber. After culturing at 37°C for 24 h, cells that had migrated or invaded to the lower chambers were fixed with 4% paraformaldehyde for 30 minutes and stained with 0.1% crystal violet for 30 minutes. Afterward, the stained cells were photographed under an inverted microscope (Leica, Germany) and counted using Image J software (version 1.53).

### Dual-Luciferase Reporter Assay

Bioinformatics analysis was applied to predict the potential binding sites of DNMBP-AS1/NHLRC3 and miR-93-5p/17-5p. Wild-type or mutant fragment of DNMBP-AS1 and NHLRC3 were constructed and inserted into the pmirGLO plasmid. Then the report plasmids and negative control or miR-93-5p/17-5p mimics were co-transfected into SW480 cells using Lipofectamine 3000 (Invitrogen, USA). After 24 h, the Dual-Luciferase Reporter Assay System (Promega, USA) was applied to detect Firefly and Renilla luciferase activity.

### Murine Xenograft Model

SW480 cells transfected with DNMBP-AS1 or control vector were harvested and suspended in PBS. Five-week-old male BALB/c nude mice (SPF Biotechnology Co., Ltd, China) were injected subcutaneously with stably transfected SW480 cells (1×10^6^), termed as vector or over-DNMBP-AS1 group (n = 3 per group). Tumor volume was monitored weekly and calculated using the formula: volume (mm^3^) = width^2^×length/2. After 5 weeks following the inoculation, the mice were euthanized and tumor samples were excised and weighted. The experiment was approved by the Animal Ethical and Welfare Committee of Zhongnan Hospital of Wuhan University and conducted following the ethics guidelines of the use of laboratory animals.

### Statistical Analysis

The data were analyzed using R (version 3.6.3) and GraphPad Prism 9 software. Differences between the two groups were assessed by unpaired Student’s t-test and those among multiple groups were assessed by one-way analysis of variance (ANOVA). The correlations were analyzed by Pearson’s test (r). The data are expressed as the mean ± standard deviation. P-value <0.05 indicated that the difference is statistically significant.

## Results

### Clinicopathological Characteristics of Included Patients

**Figure 1:**
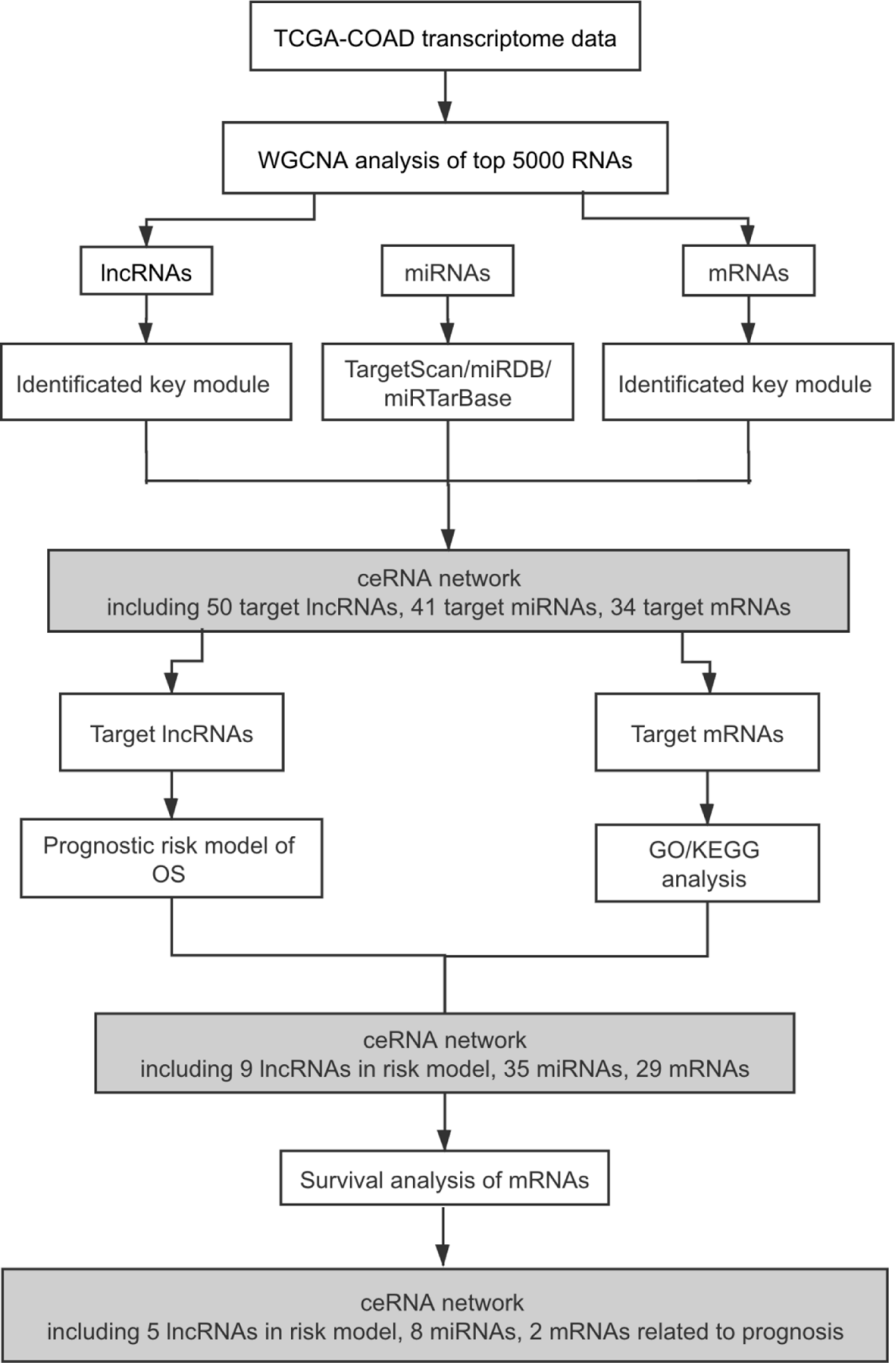
Flowchart of the study. TCGA, The Cancer Genome Atlas; COAD, colon adenocarcinoma; WGCNA, weighted gene co-expression network analysis; lncRNAs, long non-coding RNAs; miRNAs, microRNAs; mRNAs, messenger RNAs; ceRNA, competing endogenous RNA; OS, overall survival; GO, Gene Ontology; KEGG, Kyoto Encyclopedia of Genes and Genomes.

The workflow of this study is shown in **Figure 1**. Expression matrices of lncRNAs and mRNAs were collected from 320 patients, who were pathologically diagnosed as COAD. Specific clinical and pathological characteristics of all COAD patients are shown in **Table 1**, including age, gender, vital status, pathologic T stage, pathologic N stage, pathologic M stage, and tumor stage.

**Table 1:** Clinicopathological characteristics of 320 COAD patients.

Parameter	Subtype	Patients n (%)
Age	<=50	40 (12.5%)
	51-65	90 (28.1%)
	66-75	104 (32.5%)
	>75	86 (26.9%)
Gender	Male	174 (54.4%)
	Female	146 (45.6%)
Vital status	Alive	257 (80.3%)
	Dead	63 (19.7%)
T	T1	7 (2.2%)
	T2	57 (17.8%)
	T3	223 (69.7%)
	T4	33 (10.3%)
N	N0	193 (60.3%)
	N1	73 (22.8%)
	N2	54 (16.9%)
M	M0	269 (84.1%)
	M1	51 (15.9%)
Stage	Stage I	57 (17.8%)
	Stage II	129 (40.3%)
	Stage III	83 (25.9%)
	Stage IV	51 (16.0%)

COAD, colon adenocarcinoma.

### Weighted co‐Expression Network Construction and Key Module Identification

**Figure 2:**
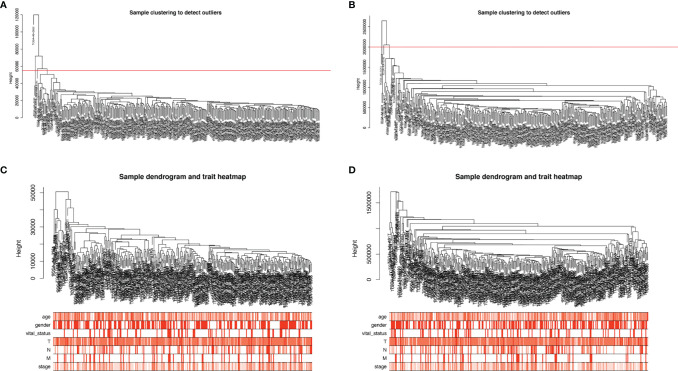
Sample cluster analysis based on RNA data from the TCGA database. **(A, B)** Sample clustering to detect outliers based on lncRNA data **(A)** and mRNA data **(B)**. The red line represents the cut-off of data filtering in the step of data preprocessing. **(C, D)** Sample dendrogram and trait heatmap based on lncRNA **(C)** and mRNA **(D)** expression data and clinical data (age, gender, vital_status, T, N, M, stage). TCGA, The Cancer Genome Atlas; lncRNA, long non-coding RNA; mRNA, messenger RNA.

**Figure 3:**
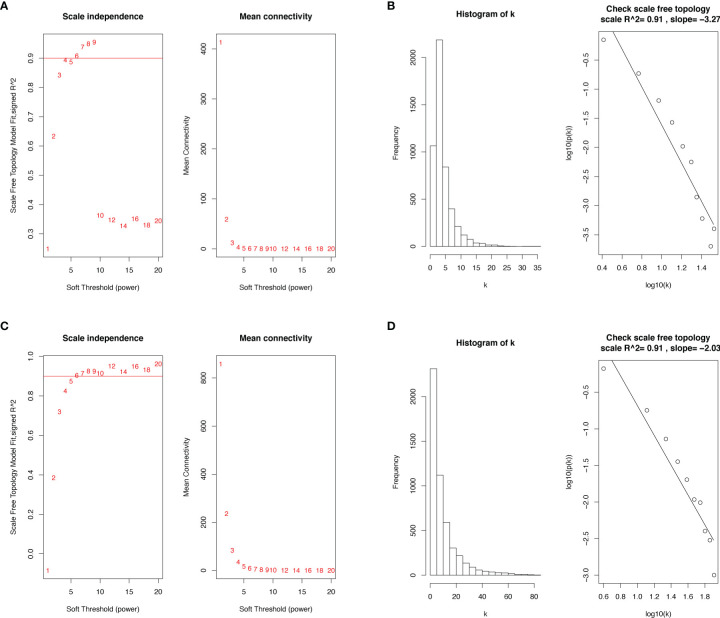
Determination of soft-thresholding power in the WGCNA. **(A)** Analysis of the scale-free topology model fit index and the mean connectivity for various soft-thresholding powers (β) in lncRNAs. **(B)** Checking the scale‐free topology when β = 4 in lncRNAs. **(C)** Analysis of the scale-free topology model fit index and the mean connectivity for various soft-thresholding powers (β) in mRNAs. **(D)** Checking the scale‐free topology when β = 6 in mRNAs. WGCNA, weighted gene co-expression network analysis; lncRNAs, long non-coding RNAs; mRNAs, messenger RNAs.

**Figure 4:**
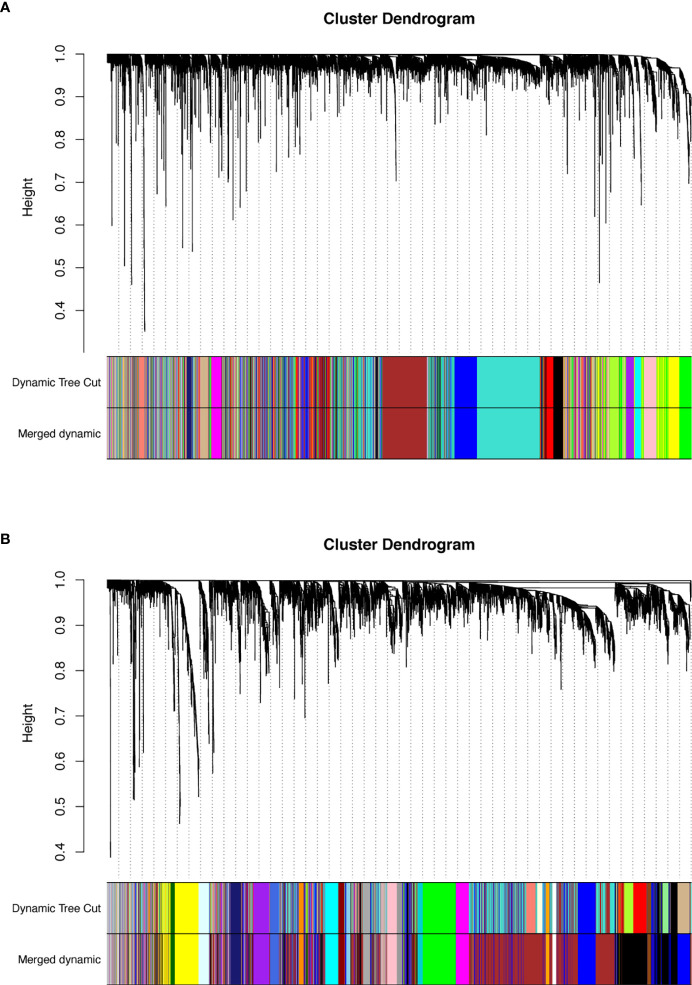
**(A)** Dendrogram of lncRNAs clustered based on a dissimilarity measure (1‐TOM). **(B)** Dendrogram of mRNAs clustered based on a dissimilarity measure (1‐TOM). The branches of the cluster dendrogram correspond to the different gene modules. Each piece of the leaves on the cluster dendrogram corresponds to a gene. lncRNAs, long non-coding RNAs; TOM, topological overlap matrix.

**Figure 5:**
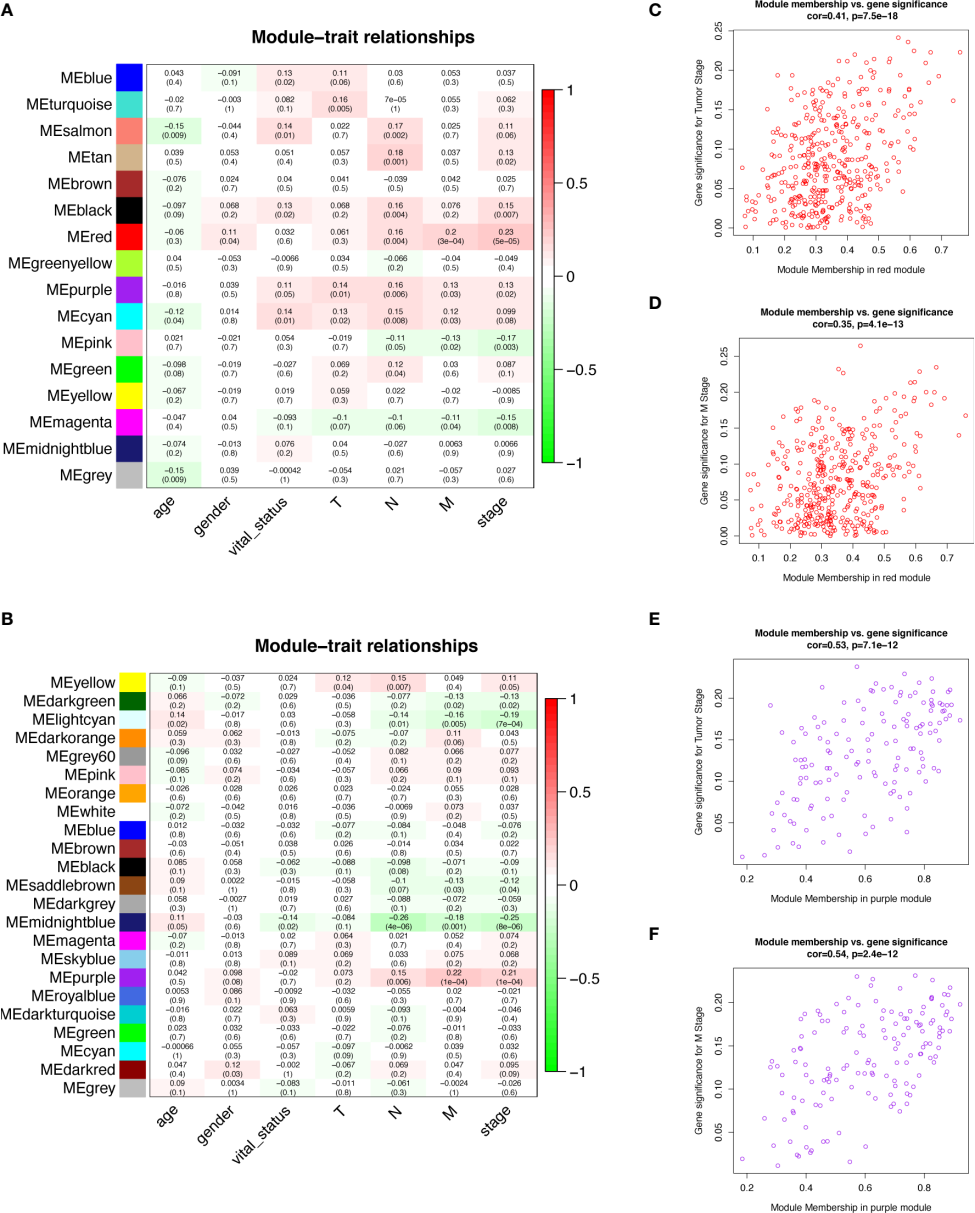
Module-trait relationships and Scatter plot of module eigengenes in key modules. **(A, B)** Heatmap of the correlation between module eigengenes and clinical traits of COAD in lncRNA data **(A)** and mRNA data **(B)**. **(C)** Scatter plot of GS for tumor stage vs. MM in the red module of lncRNAs. **(D)** Scatter plot of GS for M stage vs. MM in the red module of lncRNAs. **(E)** Scatter plot of GS for tumor stage vs. MM in the purple module of mRNAs. **(F)** Scatter plot of GS for M stage vs. MM in the purple module of mRNAs. COAD, colon adenocarcinoma; lncRNAs, long non-coding RNAs; mRNAs, messenger RNAs; GS, Gene Significance; MM, Module Membership.

We selected the top 5000 mad lncRNAs and mRNAs for co‐expression analysis with WGCNA package. The cut heights were set as 55000 (lncRNA) and 2000000 (mRNA) to remove the outliers in sampleTree (**Figures 2A, B**). The sample dendrogram and trait heatmap split the selected samples into the different clusters and provided the distribution map of clinical trait data (**Figures 2C, D**). We found that when the power was equal to 4 in lncRNAs, the scale R^2^ = 0.91 (**Figures 3A, B**); when the power was equal to 6 in mRNAs, the scale R^2^ = 0.91 (**Figures 3C, D**). Therefore, we selected β= 4 as soft-thresholding in lncRNAs, and β= 6 as soft-thresholding in mRNAs co‐expression analysis. Dendrogram of the gene modules was based on a TOM-based dissimilarity measure. Sixteen modules were screened out in lncRNAs and twenty-three modules in mRNAs were shown in different colors (**Figure 4**). The heatmap of module-trait relationships was used to select the key modules for further research. The red and purple modules were the key modules for lncRNAs and mRNAs, respectively. Both of them showed a significantly positive correlation with stage and M stage (**Figures 5A, B**). Subsequently, scatter plots of Gene Significance (GS) vs. module membership (MM) were shown in the key module. The plots revealed that MM in red module was significantly correlated with the tumor stage (cor=0.42,p=7.5e-18), and M stage (cor=0.35,p=4.1e-13). We found that MM in the purple module was significantly correlated with the tumor stage (cor=0.53,p=7.1e-12), and M stage (cor=0.54,p=2.4e-12) (**Figures 5C-F**).

### Network Analysis and Enrichment Analysis

The ceRNA network was built using 405 lncRNAs in red module and 145 mRNAs in purple module. We used miRcode database to match miRNAs with 405 lncRNAs. We got 687 lncRNA‐miRNA pairs containing 57 candidate lncRNAs and 87 candidate miRNAs. Then, based on three databases of TargetScan, miRDB, and miRTarBase, we obtained 5813 miRNA‐mRNA pairs including 87 miRNAs and 2556 candidate mRNAs. Finally, we picked out 34 target mRNAs overall after matching the 2556 candidate mRNAs with the 145 mRNAs in the purple module. Based on the above results, we constructed a lncRNA‐miRNA‐mRNA network with 50 target lncRNAs, 41 target miRNAs, and 34 target mRNAs and visualized the network using Cytoscape (**Supplementary Figure 1A**). The detailed correspondences of the original ceRNA network are shown in **Supplementary Table 2**. The GO analysis results showed that target mRNAs were chiefly enriched in steroid hormone receptor signaling pathway, negative regulation of GTPase activity, negative regulation of B cell apoptotic process, transcription factor complex, and steroid hormone receptor binding. KEGG analysis results were significantly involved in hippo signaling pathway, longevity regulating pathway - multiple species, and microRNAs in cancer (**Supplementary Figures 1B, C**).

### Prognostic Risk Model of lncRNAs

**Figure 6:**
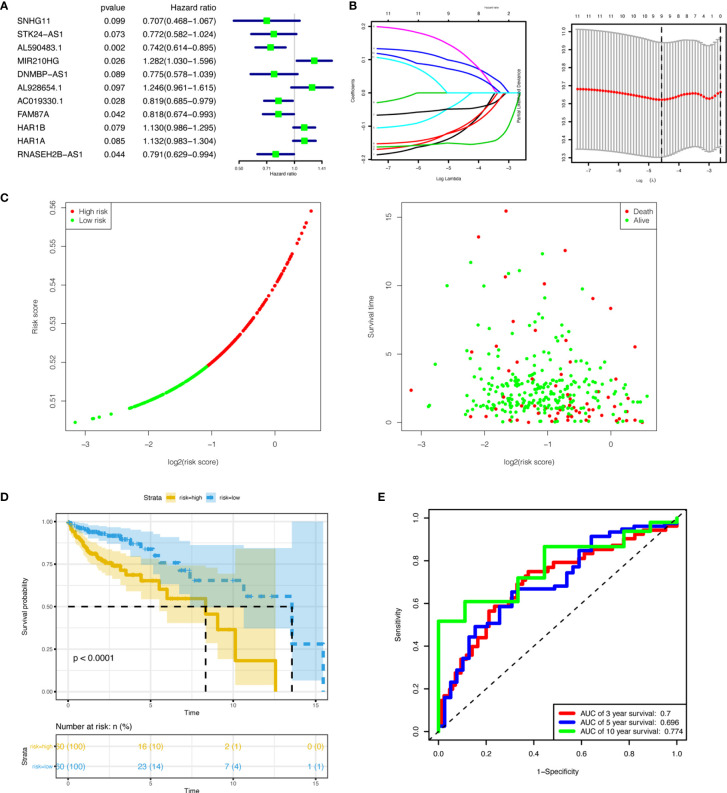
Establishment and validation of the nine-lncRNA prognostic signature. **(A)** Univariate Cox regression analyses of lncRNAs in the ceRNA network associated with overall survival. (P<0.1) **(B)** LASSO regression analysis of lncRNAs in the ceRNA network. **(C)** Performance of the prognostic signature in distinguishing patients into low-risk and high-risk groups. **(D)** Overall survival curves according to the nine-lncRNA prognostic signature of colon cancer patients with low or high risk. **(E)** ROC curves with calculated AUCs for risk prediction in 3-,5-, and 10- year. lncRNAs, long non-coding RNAs; ceRNA, competing endogenous RNA; LASSO, least absolute shrinkage and selection operator; ROC, receiver operating characteristic; AUC, area under the curve.

Univariate COX regression analyses for all 50 target lncRNAs were conducted to evaluate the associations between lncRNAs expression and OS. Eleven lncRNAs with a P-value < 0.1 in the univariate analyses were included in the LASSO regression (**Figure 6A**). Then, 9 lncRNAs (SNHG11, STK24-AS1, AL590483.1, MIR210HG, DNMBP-AS1, AL928654.1, AC019330.1, FAM87A, and HAR1A) were selected to construct a prognostic risk model for COAD after LASSO regression analysis (**Figure 6B**). The risk score of the model for OS was calculated in the following way: risk score = (-0.117 × expression level of SNHG11) + (-0.103 × expression level of STK24-AS1) + (-0.153 × expression level of AL590483.1) + (0.090 × expression level of MIR210HG) + (-0.024 × expression level of DNMBP-AS1) + (0.122 × expression level of AL928654.1) + (-0.048 × expression level of AC019330.1) + (-0.110 × expression level of FAM87A) + (0.064 × expression level of HAR1A). All 320 COAD patients were separated into high-risk and low-risk sets based on the median risk score. Vital status, survival time, and risk score levels were presented in **Figure 6C**. Survival analysis of OS showed that COAD patients with high-risk scores had a worse prognosis (P-value < 0.0001) (**Figure 6D**). The ROC curves of the risk model indicated that the values of AUC for 3-, 5-, and 10- year were 0.7, 0.696, 0.774, respectively (**Figure 6E**). From the above, the risk model based on the 9 lncRNAs revealed good performance in estimating OS. To explore the relationship between the 9 lncRNAs expression and clinical factors, we made a hierarchical cluster diagram (**Supplementary Figure 2**).

### Nomogram Construction

**Figure 7:**
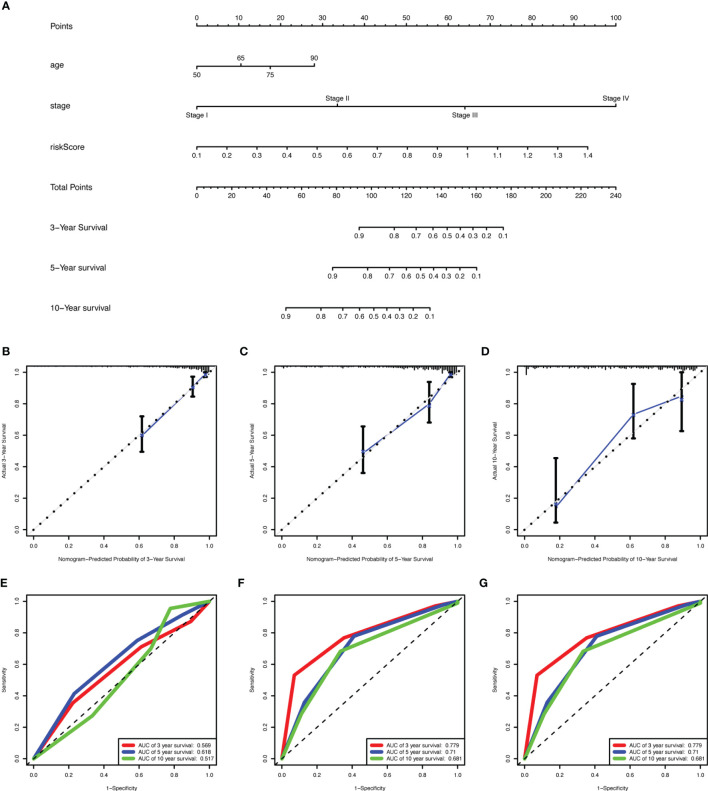
Construction of a nomogram for overall survival prediction in COAD. **(A)** The nomogram consists of age, stage, and the risk score based on the nine-lncRNA prognostic signature. **(B–D)** Calibration curves of the nomogram for the estimation of survival rates at 3- **(B)**, 5- **(C)**,10- **(D)** year. The 45-degree line indicated the actual survival outcomes. **(E–G)** ROC curves with calculated AUCs for age **(E)**, stage **(F)**, and all factors **(G)** of the nomogram in 3-, 5-,10- year. COAD, colon adenocarcinoma; lncRNA, long non-coding RNA; ROC, receiver operating characteristic; AUC, area under the curve.

We identified age, stage, and risk score as independent clinical prognostic factors by univariable and multivariable Cox regression analyses. All of the factors showed that high expression had a worse prognosis (**Table 2**). Moreover, a nomogram was built based on the three independent prognostic factors (**Figure 7A**). The C-index for the nomogram was 0.823 and the calibration curves for predicting 3-, 5-, and 10-year OS were closely matched with actual survival outcomes (**Figures 7B–D**). The values of age AUC for 3-year survival, 5-year survival, and 10-year survival were 0.569, 0.619, and 0.517, respectively. The values of stage AUC for 3-year survival, 5-year survival, and 10-year survival were 0.779, 0.71, and 0.681, respectively. The values of whole nomogram AUC for 3-year survival, 5-year survival, and 10-year survival were 0.862, 0.808, and 0.852, respectively (**Figures 7E–G**). These validate results indicated that the nomogram combined with the lncRNA prognostic signature and clinical factors provided a greater performance for OS prediction than a single factor.

**Table 2:** The selection of independent clinical prognostic factors.

Clinical characteristics	Univariable cox		Multivariable cox	
	HR (95% CI)	P-value	HR (95% CI) P	P-value
Age	1.284 [0.983-1.677]	6.65E-02	1.442 [1.104-1.882]	7.14E-03
Gender	0.940 [0.566-1.561]	8.10E-01	—	—
T	3.745 [2.216-6.329]	8.18E-07	—	—
N	2.368 [1.743-3.217]	3.49E-08	—	—
M	5.583 [3.337-9.339]	5.76E-11	—	—
Stage	2.768 [2.041-3.754]	5.78E-11	3.499 [2.529-4.840]	3.95E-14
Risk score	7.666 [3.446-17.050]	5.92E-07	13.821[5.834-32.745]	2.41E-09

HR, hazard ratio; CI, confidence interval.

### Survival-Related ceRNA Network

**Figure 8:**
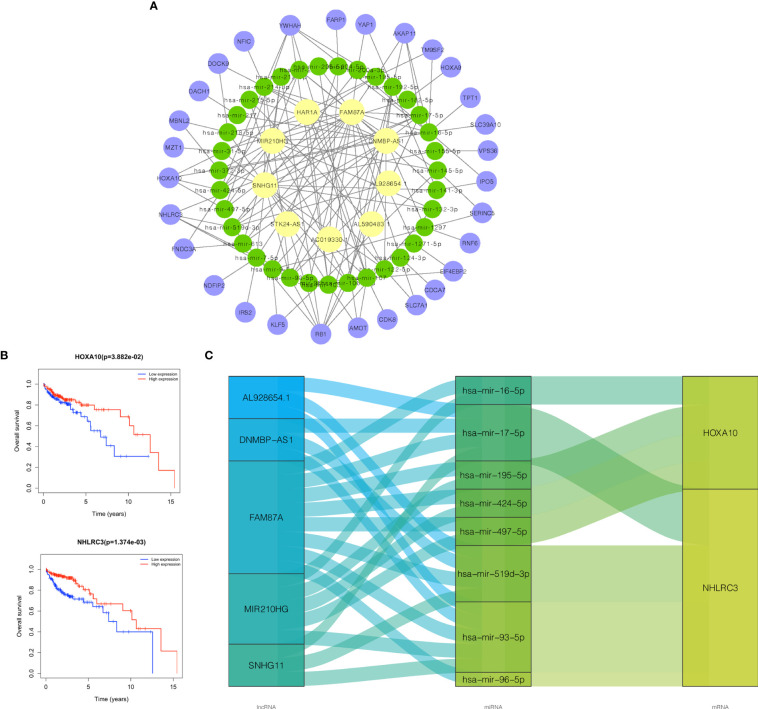
Construction of survival-related ceRNA network. **(A)** The ceRNA network constructed by 9 lncRNAs in the risk model, 35 miRNAs, and 29 mRNAs. **(B)** Kaplan‐Meier survival curves of HOXA10, NHLRC3. **(C)** The ceRNA network constructed by 5 lncRNAs in risk model, 8 miRNAs, and 2 mRNAs related to prognosis. Purple node was mRNA, green node was miRNA, and yellow node was lncRNA. ceRNA, competing endogenous RNA; lncRNAs, long non-coding RNAs; miRNAs, microRNAs; mRNAs, messenger RNAs.

Based on the lncRNAs in the prognostic risk model, the ceRNA network further shrank with 9 lncRNAs, 35 miRNAs, and 29 mRNAs (**Figure 8A**). The specific correspondences of the ceRNA network are shown in **Supplementary Table 3**. Survival analyses for 29 mRNAs was then applied and Kaplan ‐Meier curves show that HOXA10 and NHLRC3 with low expression had a worse prognosis in OS (**Figure 8B**). Therefore, we finally constructed a survival-related ceRNA network including 5 lncRNAs (SNHG11, FAM87A, DNMBP-AS1, MIR210HG, and AL928654.1), 8 miRNAs (hsa-mir-424-5p, hsa-mir-195-5p, hsa-mir-497-5p, hsa-mir-16-5p, hsa-mir-93-5p, hsa-mir-519d-3p, hsa-mir-96-5p, and hsa-mir-17-5p), and 2 mRNAs (HOXA10 and NHLRC3) (**Figure 8C**). FAM87A could form a ceRNA network with any of the miRNAs and mRNAs.

### DNMBP-AS1 and FAM87A Are Down-Regulated in Colon Cancer

**Figure 9:**
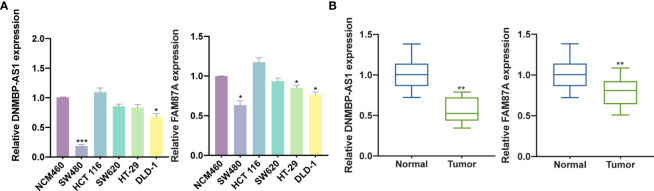
DNMBP-AS1 and FAM87A are down-regulated in colon cancer. **(A)** qRT-PCR analysis of DNMBP-AS1 and FAM87A expression in colon cancer cell lines (SW480, HCT 116, SW620, HT-29, and DLD-1) and normal human colon mucosal epithelial cell line (NCM460). **(B)** Relative expression of DNMBP-AS1 and FAM87A in colon cancer tissue samples and noncancerous tissue samples. qRT-PCR, quantitative real-time polymerase chain reaction. *P < 0.05, **P < 0.01 and ***P < 0.001.

Based on qRT-PCR results, DNMBP-AS1 and FAM87A were indicated to be down-regulated in colon cancer cells and tissues. Compared with that in NCM460 cells, DNMBP-AS1 expression was significantly down-regulated in SW480 and DLD-1, and FAM87A expression was notably down-regulated in SW480, HT-29, and DLD-1 (**Figure 9A**). Therefore, the SW480 cell line was selected for further analysis. The results of 30 pairs of human colon cancer tissues and their adjacent normal tissues showed that DNMBP-AS1 and FAM87A expression were elevated in normal tissues (**Figure 9B**).

### Over-Expression of DNMBP-AS1 and FAM87A Inhibit Colon Cancer Cell Proliferation and Metastasis

**Figure 10:**
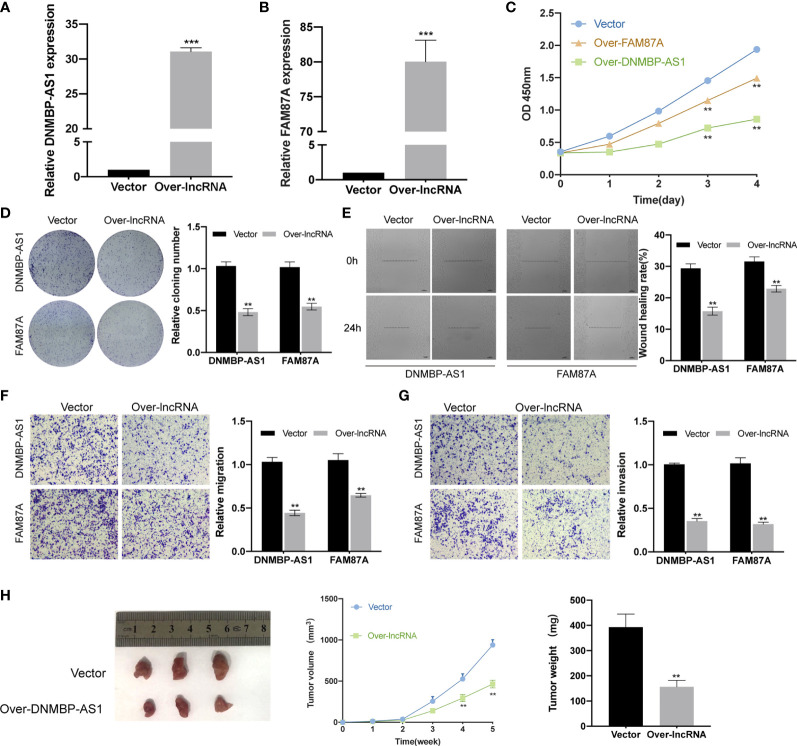
Over-expression of DNMBP-AS1 and FAM87A inhibit colon cancer cell proliferation and metastasis. **(A, B)** The expression levels of DNMBP-AS1 and FAM87A were verified after stable transfected with control vector, DNMBP-AS1 **(A)** or FAM87A **(B)** in SW480 cells. **(C)** CCK-8 assay was used to determine the viability of SW480 cells following transfection. **(D)** Colony formation in SW480 cells following transfection. **(E)** Wound healing assays in SW480 cells following transfection. Scale bars: 100 μm. **(F, G)** Transwell assays were used to assess the migration **(F)** and invasion **(G)** capacities of SW480 cells with DNMBP-AS1or FAM87A over-expression. Magnification: x100. **(H)** Representative, *in vivo* growth curve, and weight at the end points of xenograft tumors formed by subcutaneous injection of SW480 cells stable transfected with control vector or DNMBP-AS1, n=3. CCK-8, Cell Counting Kit-8. **P < 0.01 and ***P < 0.001.

To determine the function of DNMBP-AS1 and FAM87A in colon cancer, a series of assays was performed using stable transfected SW480 cells. Firstly, qRT-PCR was used to detect the expression levels of DNMBP-AS1 and FAM87A in SW480 cells transfected with control vector (vector), DNMBP-AS1 (over-DNMBP-AS1), or FAM87A (over-FAM87A). The results demonstrated that both DNMBP-AS1 and FAM87A expression significantly increased (**Figures 10A, B**). Then, CCK-8 assay and clone-formation assay were used to reveal that cell proliferative potential. Up-regulation of DNMBP-AS1 and FAM87A both significantly decreased the cell viability of SW480 cells (**Figure 10C**). Stable ectopic expression of DNMBP-AS1 and FAM87A reduced the number of clone spot than those stably transfected with the control vector (**Figure 10D**). Subsequently, wound-healing assay and transwell assay were carried out to evaluate the effects of DNMBP-AS1 and FAM87A on cell metastasis. After up-regulating lncRNA expression, both DNMBP-AS1 and FAM87A, the wound healing rates were remarkably decreased (**Figure 10E**). In transwell assay without Matrigel, migrated SW480 cells in over-lncRNA groups were less than those in the vector group (**Figure 10F**). In transwell assay with Matrigel, invaded cells were evidently decreased in over-lncRNA groups compared with that in vector group (**Figure 10G**). Finally, the animal experiment was performed to reveal the effect of stable transfected DNMBP-AS1 on cellular proliferation *in vivo*. Five weeks after subcutaneous injection, both xenograft tumors size and weight in over-DNMBP-AS1 group were obviously reduced (**Figure 10H**). On the contrary, these results suggested that over-expression of DNMBP-AS1 evidently suppressed the growth of colon cancer *in vitro* and *in vivo*.

### DNMBP-AS1 Serves as miR-93-5p/17-5p Sponges

**Figure 11:**
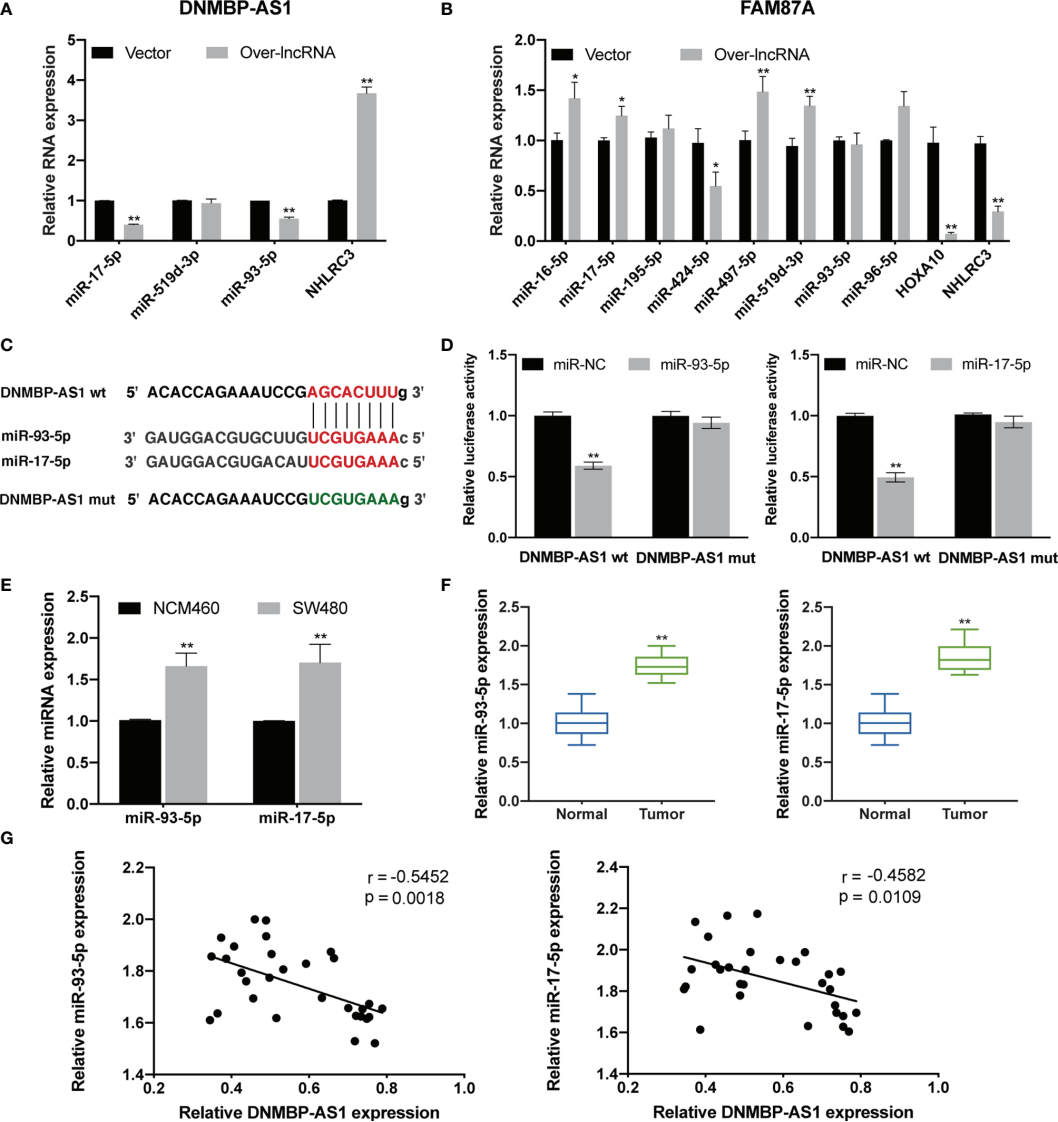
DNMBP-AS1 is a sponge of miR-93-5p/17-5p. **(A, B)** Relative expression of RNA related to DNMBP-AS1 **(A)** and FAM87A **(B)** in the final ceRNA network. **(C)** The potential binding sites of DNMBP-AS1 and miR-93-5p/17-5p were predicted. **(D)** Luciferase activity was analyzed in SW480 cells after co-transfected with DNMBP-AS1 wild-type (DNMBP-AS1 wt) or DNMBP-AS1 mutant (DNMBP-AS1 mut) and miR-93-5p mimic (miR-93-5p), miR-17-5p mimic (miR-17-5p) or miRNA negative control (miR-NC). **(E)** Relative miR-93-5p/17-5p expression in NCM460 cells and SW480 cells. **(F)** Relative miR-93-5p/17-5p expression in colon cancer tissue samples and noncancerous tissue samples. **(G)** Pearson correlation analysis determined the significant negative correlation between the levels of DNMBP-AS1 and miR-93-5p/17-5p in 30 colon cancer tissues. ceRNA, competing endogenous RNA; miRNA, microRNAs. *P < 0.05 and **P < 0.01.

Through the previous bioinformatics analysis, we predicted potential miRNAs and mRNAs that bind to DNMBP-AS1 and FAM87A. In the final ceRNA network, three miRNAs (hsa-mir-17-5p, hsa-mir-519d-3p, and hsa-mir-93-5p) and one mRNA (NHLRC3) were related to DNMBP-AS1; eight miRNAs (hsa-mir-424-5p, hsa-mir-195-5p, hsa-mir-497-5p, hsa-mir-16-5p, hsa-mir-93-5p, hsa-mir-519d-3p, hsa-mir-96-5p, and hsa-mir-17-5p), and two mRNAs (HOXA10 and NHLRC3) were related to FAM87A (**Figure 8C**). Afterward, those candidate miRNAs and mRNAs were evaluated in validation assays in SW480 cells. We found that over-expression of DNMBP-AS1 decreased the expression levels of miR-93-5p/17-5p and increased the expression level of NHLRC3, while the expression of miR-519d-3p was not affected (**Figure 11A**). As for FAM87A, the qRT-PCR results showed that the expression levels of most miRNAs were elevated and all mRNAs were significantly decreased in over-FAM87A groups (**Figure 11B**), which were not consistent with our prediction and the literature reported. Therefore, further analyses were performed to explore the effects of DNMBP-AS1 acting as ceRNA on miR-93-5p/17-5p and NHLRC3. In addition, to further excavate more miRNAs that were sponged by DNMBP-AS1 and more downstream mRNAs, we also verified the expression of miRNAs and mRNA in the secondary ceRNA network. Compared with that in the vector group, the expression of miR-106a-5p, miR-192-5p, miR-214-3p, and miR-217 were reduced, and the expression of AKAP11, RB1, NFIC, and DACH1 were increased in the over-lncRNA group (**Supplementary Figures 3A, B**). All of these results are consistent with the theoretical results of DNMBP-AS1 acting as ceRNA, so those candidate miRNAs and mRNAs are worth further vitro and vivo study.

The diagrammatic sketch of the binding sites for miR-93-5p/17-5p in DNMBP-AS1 is shown in **Figure 11C**. To find evidence of direct binding between DNMBP-AS1 and miR-93-5p/17-5p based on their complementary sequences, miRNA negative control (miR-NC), miR-93-5p mimics (miR-93-5p), or miR-17-5p mimics (miR-17-5p) were co-transfected with the wild-type or mutant DNMBP-AS1 dual-luciferase reporter, respectively. Obvious reductions in luciferase activity were observed when wild-type DNMBP-AS1 and miR-93-5p/17-5p mimics were co-transfected, but no significant change was observed in the mutant DNMBP-AS1 group (**Figure 11D**). Next, we identified miR-93-5p/17-5p were up-regulated both in colon cells (SW480) and colon tumorous tissue samples (**Figures 11E, F**). In colon cancer tissues, a negative correlation was observed between the expression of DNMBP-AS1 and miR-93-5p, and also of DNMBP-AS1 and miR-93-5p (**Figure 11G**). Taken together, this series of evidence strongly indicated that DNMBP-AS1 could act as miR-93-5p/17-5p sponges.

### miR-93-5p/17-5p Reverse the Inhibition Function of DNMBP-AS1 Over-Expression in Colon Cancer Cells

**Figure 12:**
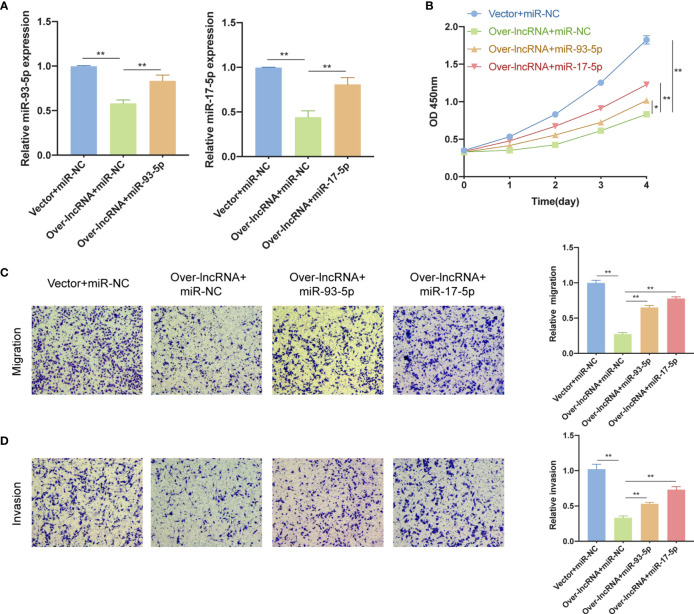
miR-93-5p/17-5p reverse the inhibition effects of DNMBP-AS1 over-expression in colon cancer cells. **(A)** Relative miR-93-5p/17-5p expression in SW480 cells transfected with control vector and miRNA negative control (Vector + miR-NC), DNMBP-AS1 and miRNA negative control (Over-lnc + miR-NC), and DNMBP-AS1 and miR-93-5p/17-5p mimics (Over-lnc + miR-93-5p/17-5p). **(B)** CCK-8 assay was used to determine the viability of SW480 cells following transfection. **(C, D)** Transwell assays were used to assess the migration **(C)** and invasion **(D)** capacities of SW480 cells following transfection. Magnification: x100. miRNA, microRNAs; CCK-8, Cell Counting Kit-8. *P < 0.05 and **P < 0.01.

qRT-PCR analysis was performed to further confirm that over-expression of DNMBP-AS1 could decrease the miR-93-5p/17-5p expression levels (**Figure 12A**) while miR-93-5p/17-5p expression levels could markedly rise when transfected with miR-93-5p/17-5p mimics (**Figure 12A**). To confirm whether DNMBP-AS1 exerts its biological function by sponging miR-93-5p/17-5p, the CCK-8 assay and transwell assay were conducted for rescue experiments. We observed that when DNMBP-AS1 and miR-93-5p/17-5p mimics were co-transfected, the reduced cell viability mediated by DNMBP-AS1 over-expression could be reversed (**Figure 12B**). The number of migrated cells and invaded cells were elevated in DNMBP-AS1 and miR-93-5p/17-5p co-transfected groups compared to those in DNMBP-AS1 and miR-NC co-transfected group (**Figures 12C, D**). This evidence suggested that DNMBP-AS1 affected colon cancer cell proliferation and metastasis by sponging miR-93-5p/17-5p.

### DNMBP-AS1 Promotes NHLRC3 Expression Through Sponging miR-93-5p/17-5p

**Figure 13:**
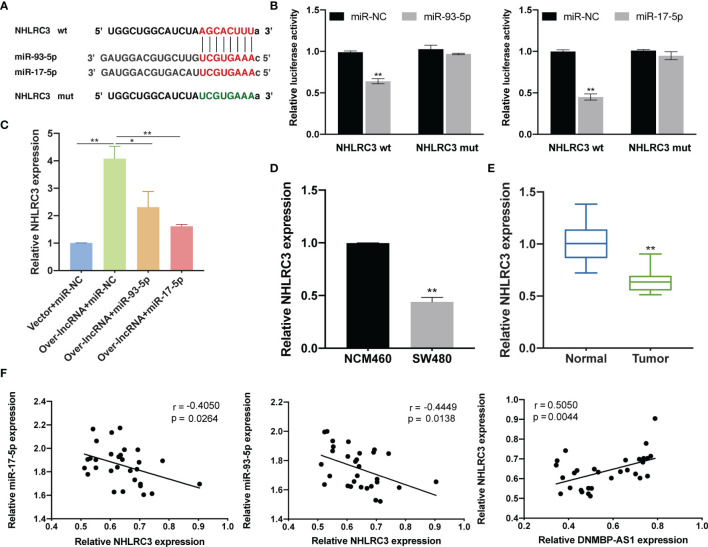
DNMBP-AS1 promotes NHLRC3 expression through sponging miR-93-5p/17-5p. **(A)** The potential binding sites of NHLRC3 and miR-93-5p/17-5p were predicted. **(B)** Luciferase activity was analyzed in SW480 cells after co-transfected with Luciferase activity was analyzed in SW480 cells after co-transfected with NHLRC3 wild-type (NHLRC3 wt) or NHLRC3 mutant (NHLRC3 mut) and miR-93-5p mimics (miR-93-5p), miR-17-5p mimics (miR-17-5p) or miRNA negative control (miR-NC). **(C)** Relative NHLRC3 expression in SW480 cells transfected with control vector and miRNA negative control (Vector + miR-NC), DNMBP-AS1 and miRNA negative control (Over-lnc + miR-NC), and DNMBP-AS1 and miR-93-5p/17-5p mimic (Over-lnc + miR-93-5p/17-5p). **(D)** Relative NHLRC3 expression in NCM460 cells and SW480 cells. **(E)** Relative NHLRC3 expression in colon cancer tissue samples and noncancerous tissue samples. **(F)** Pearson correlation analysis determined the significant negative correlation between the levels of NHLRC3 and miR-93-5p/17-5p and positive correlation between the levels of DNMBP-AS1 and NHLRC3 in 30 colon cancer tissues. miRNA, microRNAs. *P < 0.05 and **P < 0.01.

The bioinformatics prediction results demonstrated that there were binding sites between miR-93-5p/17-5p and 3′UTR of NHLRC3 (**Figure 13A**). The results of the dual luciferase reporter assay revealed that luciferase activity was reduced after co-transfection of wild-type NHLRC3 and miR-93-5p/17-5p mimics, while no reduction in luciferase activity was observed in the mutant group (**Figure 13B**). In order to further explore the interactions among DNMBP-AS1, miR-93-5p/17-5p, and NHLRC3, we detected NHLRC3 expression in co-transfected miR-NC, miR-93-5p, or miR-17-5p with vector or over-lncRNA. We found that in the cells transfected with DNMBP-AS1 and miRNA negative control (Over-lncRNA + miR-NC), the expression of NHLRC3 was up-regulated in SW480 cells (**Figure 13C**). On the contrary, in the cells transfected with DNMBP-AS1 and miR-93-5p/17-5p mimic (Over-lncRNA + miR-93-5p/17-5p), the expression of NHLRC3 was down-regulated compared with Over-lncRNA + miR-NC group (**Figure 13C**). The mRNA expression of NHLRC3 was examined by qRT-PCR, NHLRC3 were down-expressed both in colon cells and colon cancer tissues (**Figures 13D, E**). Pearson correlation results manifested that NHLRC3 expression were negatively correlated with miR-93-5p/17-5p and that was positively correlated with DNMBP-AS1 in 30 pairs of human colon samples (**Figure 13F**). All of the results illustrated that DNMBP-AS1 competitively bound to miR-93-5p/17-5p and acted as ceRNA to boost NHLRC3 expression.

## Discussion

In the present study, top mad 5000 lncRNAs and top mad 5000 mRNAs were used to perform WGCNA analysis. We then selected 405 lncRNAs in the red module and 145 mRNAs in purple module to build the original ceRNA network, which contained 50 target lncRNAs, 41 target miRNAs, and 34 target mRNAs. The results of GO and KEGG analyses showed that target mRNAs were significantly enriched in hippo signaling pathway. It has been identified that the hippo signaling pathway could regulate multiple biological processes and was a key cancer signaling network in humans (15). Besides, a study has shown that the hippo signaling pathway could inhibit the proliferation of stem cells and progenitor cells (16). We selected 9 lncRNAs (SNHG11, STK24-AS1, AL590483.1, MIR210HG, DNMBP-AS1, AL928654.1, AC019330.1, FAM87A, and HAR1A) to construct a prognostic risk model for COAD. The secondary ceRNA network included 9 lncRNAs in the risk model, 35 miRNAs, and 29 mRNAs. Survival analyses of the 29 mRNAs have shown HOXA10 and NHLRC3 were identified as crucial prognostic factors. Finally, we constructed the last ceRNA network related to survival including 5 lncRNAs, 8 miRNAs, and 2 mRNAs. As relevant experimental studies of the effect of FAM87A and DNMBP-AS1 on colon cancer progression have not been published, we performed further assays to research function and underlying mechanisms of these lncRNAs in colon cancer. Our study elucidated that DNMBP-AS1 and FAM87A were down-regulated in CC cells and tissues, and that over-expression of DNMBP-AS1 and FAM87A inhibited CC cells proliferation and migration. Mechanistically, DNMBP-AS1 up-regulated the expression of NHLRC3 through sponging miR-93-5p/17-5p in CC.

LncRNA SNHG11 (also known as small nucleolar RNA host gene 11) is found to facilitate cell proliferation and migration in a variety of cancers, such as lung cancer (17), glioma (18), and hepatocellular carcinoma (HCC) (19). High SNHG11 expression promotes proliferation and metastasis in colorectal cancer (CRC) by targeting the hippo pathway (20). SNHG11, which is highly expressed in CRC, promotes the stability of c-myc by binding to IGF2BP1, and c-myc in turn could act as a transcription factor to promote the expression of SNHG11 (21). High expression AL590483.1 has been found to be correlated with the good prognosis of patients with COAD (22). AL590483.1 was included in a 15-lncRNA signature to predict the OS of patients with CRC, and it may function as a ceRNA to regulate the p53 and Wnt signaling pathways and thus regulate tumor progression (23). There are also some studies on MIR210HG in ceRNA network. For example, the upregulation of MIR210HG could promote cervical cancer progression by regulating miR-503-5p/TRAF4 axis (24). Moreover, MIR210HG promotes tumor metastasis *via* binding miR-1226-3p to regulate MUC1-C and EMT pathway in invasive breast cancer (25). MIR210HG also sponges miR-503 to promote osteosarcoma cell invasion and metastasis (26). FAM87A (also known as family with sequence similarity 87 member A) could regulate the progression of tongue squamous cell carcinoma (TSCC) through ceRNA network, and high expression of FAM87A is associated with poor prognosis of TSCC patients (27). Highly accelerated region 1A (HAR1A), also known as HAR1F and LINC00064, shows that decreased HAR1A indicates advanced histological grade, advanced TNM stage, and poor prognosis in HCC (28). Up-regulation of HAR1A is thought to improve the survival of diffusing glioma patients who received chemotherapy and radiotherapy (29). There have been no studies on lncRNA STK24-AS1, DNMBP-AS1, AL928654.1, and AC019330.1, which were, for the first time, reported in our article.

A study uncovered that highly expressed miR-17-5p promotes gastric cancer proliferation and invasion by negatively regulating RUNX3 (30). Long non-coding RNA HOTAIR up-regulates the expression of p21 *via* miR-17-5p to promoted myeloid differentiation in acute myeloid leukemia (31). Hsa_circ_0107593 could directly sponge miR-93-5p to suppress the progression of cervical cancer (32). cESRP1 could enhance chemosensitivity in small cell lung cancer by sponging miR-93-5p and thereby inhibiting the TGF-β pathway (33).

Homeobox A10 (HOXA10) may function in fertility, embryo viability, and regulation of hematopoietic lineage commitment. MiR-27b-3p promotes cell migration and invasion in CRC by targeting HOXA10 (34). In addition, LINC00355 and LINC00461 act as a miR-195 sponge to promote cell proliferation, invasion, and migration to up-regulate HOXA10 in head, neck squamous cell carcinoma, and lung adenocarcinoma, respectively (35, 36). NHL repeat containing 3(NHLRC3), is involved in the development of a prognostic model based on seven-gene signature in CRC (37).

## Conclusion

In summary, we identified that 9 lncRNAs (SNHG11, STK24-AS1, AL590483.1, MIR210HG, DNMBP-AS1, AL928654.1, AC019330.1, FAM87A, and HAR1A) in the prognostic risk model could be used to predict survival rates, and that 2 mRNAs (HOXA10 and NHLRC3) are related to the prognosis of COAD. We indicated that DNMBP-AS1 inhibited the growth and metastasis of CC through a novel regulatory axis formed by DNMBP-AS1/miR-93-5p/17-5p/NHLRC3. This study constructed a survival-related lncRNA-miRNA-mRNA network and provided a new direction for exploring the molecular mechanisms of colon cancer progression.

## Data Availability Statement

The datasets presented in this study can be found in online repositories. The names of the repository/repositories and accession number(s) can be found in the article/**Supplementary Material**.

## Ethics Statement

The studies involving human participants were reviewed and approved by ethics committee of the Zhongnan Hospital of Wuhan University. The patients/participants provided their written informed consent to participate in this study. The animal study was reviewed and approved by Animal Ethical and Welfare Committee of Zhongnan Hospital of Wuhan University. Written informed consent was obtained from the individual(s) for the publication of any potentially identifiable images or data included in this article.

## Author Contributions

LY and XL conceived the study and drafted the manuscript. LY, TY and HW performed data mining, acquisition and analysis. LY, TD and XF refined the experimental scheme and conducted the experiments. LS collected tissue samples. XL and MF revised the manuscript critically for important intellectual content. All authors read and approved the final manuscript.

## Funding

This work was supported by National Natural Science Foundation of China (81770283, 82070302), the Clinical Medical Research Center of Peritoneal Cancer of Wuhan (2015060911020462). Funding agency did not participate in the design of the study and collection, analysis, and interpretation of data and in writing the manuscript.

## Conflict of Interest

The authors declare that the research was conducted in the absence of any commercial or financial relationships that could be construed as a potential conflict of interest.

## Publisher’s Note

All claims expressed in this article are solely those of the authors and do not necessarily represent those of their affiliated organizations, or those of the publisher, the editors and the reviewers. Any product that may be evaluated in this article, or claim that may be made by its manufacturer, is not guaranteed or endorsed by the publisher.
