# Supplementary figures and images for: DNMBP-AS1 Regulates NHLRC3 Expression by Sponging miR-93-5p/17-5p to Inhibit Colon Cancer Progression
Source: Front Oncol. 2022 Apr 27;12:765163. doi: 10.3389/fonc.2022.765163 (PMC9092830; doi:10.3389/fonc.2022.765163)

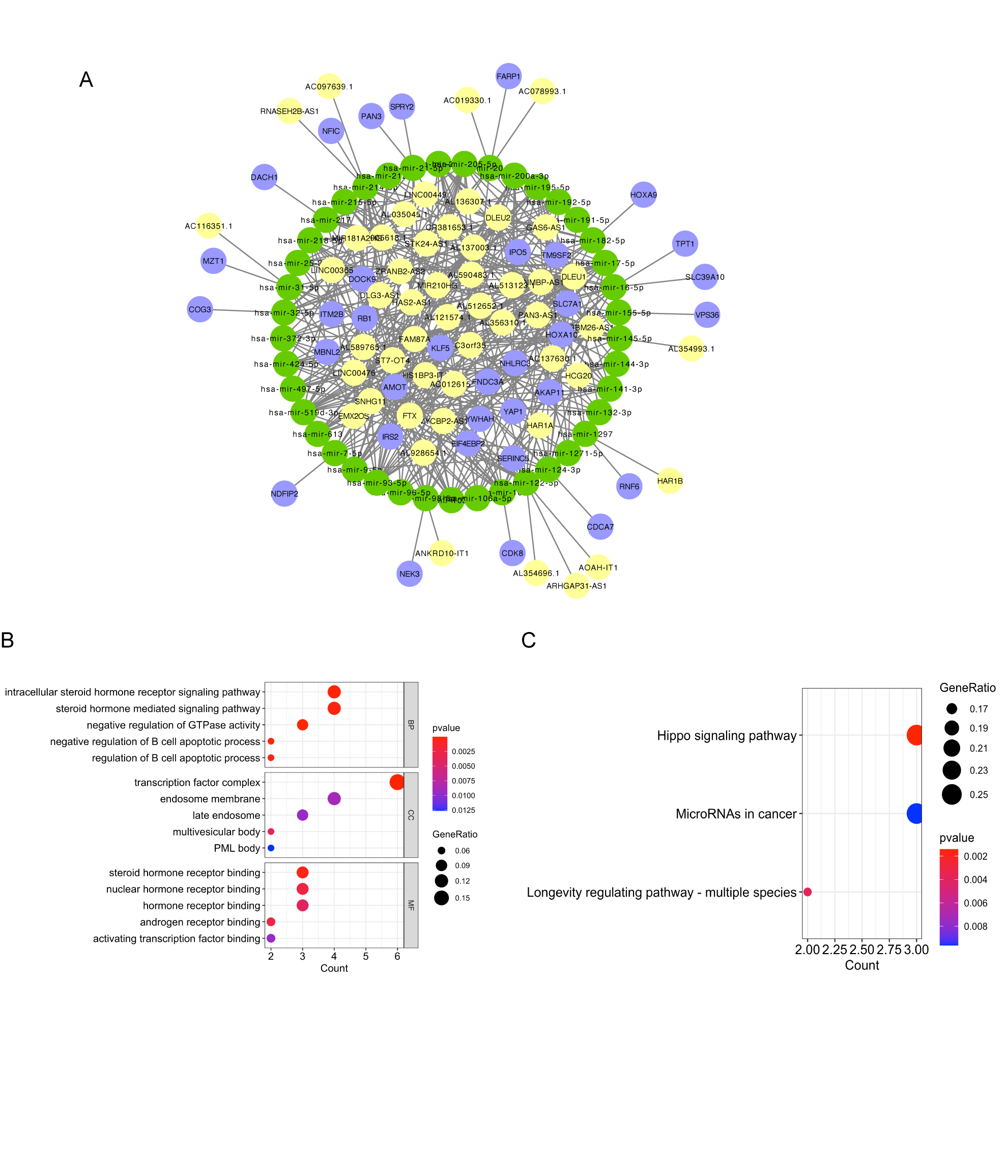

Supplement: Supplementary file 2 [file DataSheet_2.zip › Supplementary Figure 1.tif]

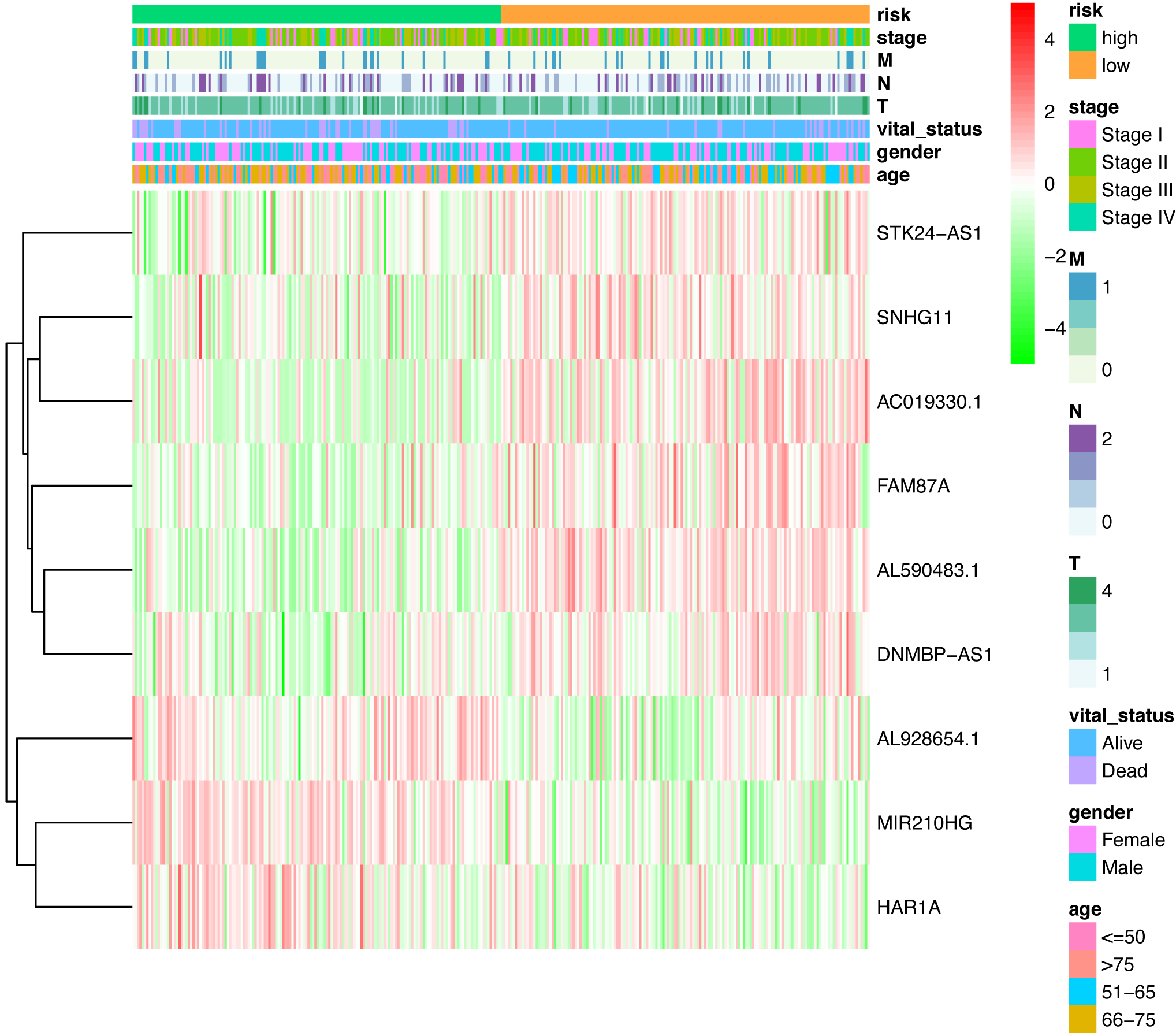

Supplement: Supplementary file 2 [file DataSheet_2.zip › Supplementary Figure 2.tif]

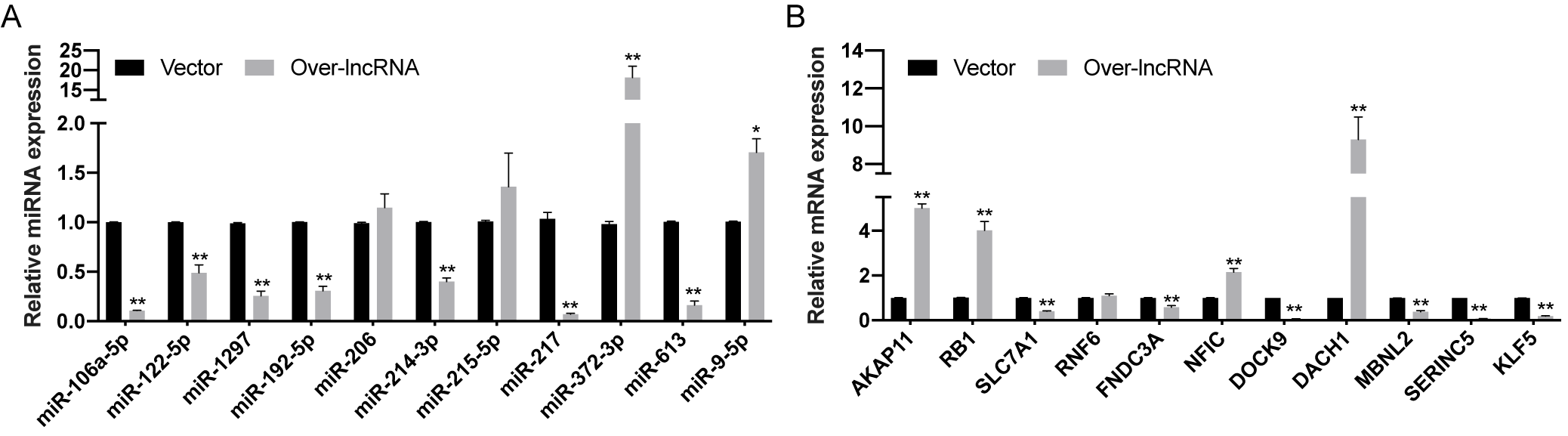

Supplement: Supplementary file 2 [file DataSheet_2.zip › Supplementary Figure 3.tif]
