# Supplementary material for: DNMBP-AS1 Regulates NHLRC3 Expression by Sponging miR-93-5p/17-5p to Inhibit Colon Cancer Progression
Source: Front Oncol. 2022 Apr 27;12:765163. doi: 10.3389/fonc.2022.765163 (PMC9092830; doi:10.3389/fonc.2022.765163)
Supplement: Supplementary file 3 [file DataSheet_3.docx]

Supplementary Material

# Supplementary Figures and Tables

## Supplementary Figures

**Supplementary Figure 1.** Construction of ceRNA network and enrichment analyses of genes included in the ceRNA network. (A) The ceRNA network constructed by 50 lncRNAs in the red module, 41 miRNAs, and 34 mRNAs in the purple module. Purple node was mRNA, green node was miRNA, and yellow node was lncRNA. (B-C) GO analysis (B) and KEGG pathway analysis (C) of 34 mRNAs.

ceRNA, competing endogenous RNA; lncRNAs, long non-coding RNAs; miRNAs, microRNAs; mRNAs, messenger RNAs; GO, Gene Ontology; KEGG, Kyoto Encyclopedia of Genes and Genomes

**Supplementary Figure 2.** Heatmap of clinical traits and the expression level of lncRNAs in the risk model.

lncRNAs, long non-coding RNAs

**Supplementary Figure 3.** Relative expression of miRNA (A) and mRNA (B) related to DNMBP-AS1 in the secondary ceRNA network.

miRNAs, microRNAs; mRNAs, messenger RNAs; ceRNA, competing endogenous RNA

## Supplementary Tables

**Supplementary Table 1.** Primers used in this study.

| Primer name | Sequence（5' to 3'） |
| --- | --- |
| miR-106a-5p-RT | GTCGTATCGACTGCAGGGTCCGAGGTATTCGCAGTCGATACGACCTACCT |
| miR-106a-5p-F | CGGCAAAAGTGCTTACAGTGC |
| miR-122-5p-RT | GTCGTATCGACTGCAGGGTCCGAGGTATTCGCAGTCGATACGACCAAACA |
| miR-122-5p-F | CGGCTGGAGTGTGACAATGG |
| miR-1297-RT | GTCGTATCGACTGCAGGGTCCGAGGTATTCGCAGTCGATACGACCACCTG |
| miR-1297-F | CGGCCGGCTTCAAGTAATT |
| miR-17-5p-RT | GTCGTATCGACTGCAGGGTCCGAGGTATTCGCAGTCGATACGACCTACCT |
| miR-17-5p-F | GGCCAAAGTGCTTACAGTGC |
| miR-192-5p-RT | GTCGTATCGACTGCAGGGTCCGAGGTATTCGCAGTCGATACGACGGCTGT |
| miR-192-5p-F | CCGGCCTGACCTATGAATTG |
| miR-206-RT | GTCGTATCGACTGCAGGGTCCGAGGTATTCGCAGTCGATACGACCCACAC |
| miR-206-F | CCGGCTGGAATGTAAGGAAGT |
| miR-214-3p-RT | GTCGTATCGACTGCAGGGTCCGAGGTATTCGCAGTCGATACGACACTGCC |
| miR-214-3p-F | GGCACAGCAGGCACAGACA |
| miR-215-5p-RT | GTCGTATCGACTGCAGGGTCCGAGGTATTCGCAGTCGATACGACGTCTGT |
| miR-215-5p-F | CCGGCATGACCTATGAATTG |
| miR-217-5p-RT | GTCGTATCGACTGCAGGGTCCGAGGTATTCGCAGTCGATACGACTCCAAT |
| miR-217-5p-F | CGGCTACTGCATCAGGAACTG |
| miR-372-3p-RT | GTCGTATCGACTGCAGGGTCCGAGGTATTCGCAGTCGATACGACACGCTC |
| miR-372-3p-F | GGCAAAGTGCTGCGACATTT |
| miR-519d-3p-RT | GTCGTATCGACTGCAGGGTCCGAGGTATTCGCAGTCGATACGACCACTCT |
| miR-519d-3p-F | GGCCAAAGTGCCTCCCTTT |
| miR-613-RT | GTCGTATCGACTGCAGGGTCCGAGGTATTCGCAGTCGATACGACGGCAAA |
| miR-613-F | CCGGCAGGAATGTTCCTTC |
| miR-9-5p-RT | GTCGTATCGACTGCAGGGTCCGAGGTATTCGCAGTCGATACGACTCATAC |
| miR-9-5p-F | CCGGCTCTTTGGTTATCTAGCT |
| miR-93-5p-RT | GTCGTATCGACTGCAGGGTCCGAGGTATTCGCAGTCGATACGACCTACCT |
| miR-93-5p-F | GCCAAAGTGCTGTTCGTGC |
| common-R | ACTGCAGGGTCCGAGGTATT |
| AKAP11-F | TGCAGTGTAACAGCAGAGGA |
| AKAP11-R | TCCAAAGAAACTGCAGCTAAATCC |
| DACH1-F | GAGACCCCGCTTTCTACACC |
| DACH1-R | ATGGCAACTTTCAACAGCCC |
| DOCK9-F | CTTGGGATGGGCAGGCATTA |
| DOCK9-R | CTTGGGCTCCAGATTCGGTT |
| FNDC3A-F | GCCTTTAGCCACCAGAACCT |
| FNDC3A-R | TTCTTGGGAGAGGGGACCTT |
| HOXA10-F | ATTGTTACCACTGGTAACATGT |
| HOXA10-R | AGGCTTCTGTGAATTTCAGAAA |
| KLF5-F | CCACCTCCATCCTATGCTGC |
| KLF5-R | TGGATGCGTCGTTTCTCCAA |
| MBNL2-F | TGAGCTTCATACCCCACCAAA |
| MBNL2-R | ACAGGCAACTGGATGGTGAG |
| NFIC-F | CAGCACTGGCCTCAGAAGAA |
| NFIC-R | AGTGTAGTAATCGCCGCCAG |
| NHLRC3-F | GTTGGCCTAAGCACCCAGAA |
| NHLRC3-R | CCGTGATCCAGACGGATTGT |
| RB1-F | CAAAGGACCGAGAAGGACCA |
| RB1-R | TGGAAGGCTGAGGTTGCTTG |
| RNF6-F | AAGAGCGTCTCCACAGAGAA |
| RNF6-R | GTTGTTCCTTGACGCCATCT |
| SERINC5-F | CAACAACCGTGGCTCACAAG |
| SERINC5-R | CACGGCAGAATATCCCACCA |
| SLC7A1-F | CATCGCCTACTTTGGGGTGT |
| SLC7A1-R | TAACCCGAGGCATGGGAAAC |
| GAPDH-F | GGAGTCCACTGGCGTCTTCA |
| GAPDH-R | GTCATGAGTCCTTCCACGATACC |
| U6-F | CGCTTCGGCAGCACATATAC |
| U6-R | TCACGAATTTGCGTGTCATC |

RT, reverse transcription; F, Forward; R, Reverse

**Supplementary Table 2.** Specific miRNAs may target lncRNAs in the red module and target mRNAs in the purple module.

| lncRNAs | miRNAs | mRNAs |
| --- | --- | --- |
| SNHG11,LINC00476,DLEU1,FAM87A,ST7-OT4,AL137003.1,HAR1A,AL589765.1,AC137630.1,HAS2-AS1 | hsa-mir-103a-3p | AMOT,YWHAH |
| SNHG11,LINC00476,DLEU1,LINC00449,AL137003.1,RBM26-AS1,DNMBP-AS1,MIR210HG | hsa-mir-106a-5p | AKAP11,RB1 |
| SNHG11,LINC00476,DLEU1,FAM87A,ST7-OT4,AL137003.1,HAR1A,AL589765.1,AC137630.1,HAS2-AS1 | hsa-mir-107 | AMOT,CDK8,YWHAH |
| SNHG11,LINC00476,DLEU1,AL512652.1,ST7-OT4,HAR1A,DNMBP-AS1,AOAH-IT1,FTX,DLEU2,MYCBP2-AS1,ARHGAP31-AS1,AL354696.1,MIR210HG | hsa-mir-122-5p | SLC7A1 |
| DLEU1,FAM87A,AC012615.1,RBM26-AS1,EMX2OS,DLEU2,GAS6-AS1,MYCBP2-AS1,CR381653.1 | hsa-mir-124-3p | CDCA7,SLC7A1 |
| DLEU1,FAM87A,ST7-OT4,DLEU2,GAS6-AS1 | hsa-mir-1271-5p | EIF4EBP2 |
| LINC00476,C3orf35,DNMBP-AS1,HCG20,HAR1B,HAS2-AS1 | hsa-mir-1297 | RNF6 |
| SNHG11,LINC00476,DLEU1,AL137003.1,MIR210HG | hsa-mir-132-3p | RB1 |
| SNHG11,DLEU1,FAM87A,AL512652.1,ST7-OT4,AL137003.1,AL513123.1,FTX,DLEU2 | hsa-mir-141-3p | IPO5,YAP1 |
| LINC00476,HCG20,FTX,DLEU2 | hsa-mir-144-3p | FNDC3A,HOXA10 |
| DLEU1,FAM87A,AL137003.1,MIR181A2HG,AL354993.1,FTX,CR381653.1,AL589765.1,MIR210HG,HAS2-AS1,PAN3-AS1 | hsa-mir-145-5p | KLF5,SERINC5 |
| SNHG11,LINC00476,DLEU1,LINC00365,MYCBP2-AS1 | hsa-mir-155-5p | VPS36 |
| LINC00476,DLEU1,FAM87A,ST7-OT4,MIR181A2HG,FTX,AL589765.1,MIR210HG | hsa-mir-16-5p | AKAP11,HOXA10,SLC39A10,TM9SF2,TPT1,YWHAH |
| SNHG11,LINC00476,DLEU1,FAM87A,C3orf35,LINC00449,AL512652.1,AL137003.1,LINC00365,RBM26-AS1,DNMBP-AS1,ZRANB2-AS2,AL928654.1,PAN3-AS1 | hsa-mir-17-5p | AKAP11,NHLRC3,RB1 |
| DLEU1,ST7-OT4,AL137003.1,EMX2OS  FTX,DLEU2,GAS6-AS1,MIR210HG | hsa-mir-182-5p | HOXA9 |
| CR381653.1 | hsa-mir-191-5p | SLC7A1 |
| LINC00365,DNMBP-AS1,ZRANB2-AS2,FTX,AL136307.1,AC137630.1,AL121574.1,AL590483.1 | hsa-mir-192-5p | RB1 |
| LINC00476,DLEU1,FAM87A,ST7-OT4,MIR181A2HG,FTX,AL589765.1,MIR210HG | hsa-mir-195-5p | AKAP11,HOXA10,TM9SF2,YWHAH |
| SNHG11,DLEU1,FAM87A,AL512652.1,ST7-OT4,AL137003.1,AL513123.1,FTX,DLEU2 | hsa-mir-200a-3p | IPO5,YAP1 |
| DLEU1,C3orf35,STK24-AS1,ZRANB2-AS2,CR381653.1,AC019330.1,AC078993.1 | hsa-mir-204-5p | FARP1 |
| LINC00476,DLEU1,FAM87A,C3orf35,AL512652.1,ST7-OT4,AL137003.1,MIR181A2HG,EMX2OS,FTX,DLEU2,DLG3-AS1,GAS6-AS1 | hsa-mir-205-5p | AMOT |
| DLEU1,FAM87A,C3orf35,AL512652.1,AL137003.1,AL356310.1,RBM26-AS1,DNMBP-AS1,DLEU2,MIR210HG | hsa-mir-206 | FNDC3A |
| SNHG11,LINC00476,DLEU1,AL137003.1,MIR210HG | hsa-mir-212-3p | RB1 |
| SNHG11,LINC00476,DLEU1,FAM87A,AC012615.1,AC097639.1,ST7-OT4,LINC00365,HAR1A,DNMBP-AS1,FTX,DLEU2,DLG3-AS1,RNASEH2B-AS1,GAS6-AS1,MIR210HG,AC005618.1 | hsa-mir-214-3p | DOCK9,NFIC |
| LINC00365,DNMBP-AS1,ZRANB2-AS2,FTX,AL136307.1,AC137630.1,AL121574.1,AL590483.1 | hsa-mir-215-5p | RB1 |
| LINC00476,LINC00365,FTX,HS1BP3-IT1,CR381653.1 | hsa-mir-21-5p | PAN3,SPRY2 |
| DLEU1,LINC00449,AL137003.1,RBM26-AS1,DNMBP-AS1,HS1BP3-IT1,AL589765.1,AC005618.1 | hsa-mir-217 | DACH1 |
| SNHG11,FAM87A,MIR181A2HG,FTX,DLG3-AS1,HS1BP3-IT1,GAS6-AS1,CR381653.1,MIR210HG,HAS2-AS1 | hsa-mir-218-5p | DOCK9,MBNL2 |
| DLEU1,ST7-OT4,AL035045.1,LINC00365,ZRANB2-AS2,DLEU2,GAS6-AS1 | hsa-mir-25-3p | ITM2B |
| SNHG11,DLEU1,FAM87A,C3orf35,AC116351.1,ST7-OT4,AL137003.1,EMX2OS | hsa-mir-31-5p | MZT1 |
| DLEU1,ST7-OT4,AL035045.1,LINC00365,ZRANB2-AS2,DLEU2,GAS6-AS1 | hsa-mir-32-5p | COG3,DOCK9,ITM2B |
| SNHG11,LINC00476,DLEU1,LINC00449,AL137003.1,RBM26-AS1,DNMBP-AS1,MIR210HG | hsa-mir-372-3p | MBNL2 |
| LINC00476,DLEU1,FAM87A,ST7-OT4,MIR181A2HG,FTX,AL589765.1,MIR210HG | hsa-mir-424-5p | HOXA10,YWHAH |
| LINC00476,DLEU1,FAM87A,ST7-OT4,MIR181A2HG,FTX,AL589765.1,MIR210HG | hsa-mir-497-5p | HOXA10,YWHAH |
| SNHG11,LINC00476,DLEU1,FAM87A,C3orf35,LINC00449,AL512652.1,AL137003.1,LINC00365,RBM26-AS1,DNMBP-AS1,ZRANB2-AS2,AL928654.1,PAN3-AS1 | hsa-mir-519d-3p | NHLRC3,RB1 |
| DLEU1,FAM87A,C3orf35,AL512652.1,AL137003.1,AL356310.1,RBM26-AS1,DNMBP-AS1,DLEU2,MIR210HG | hsa-mir-613 | FNDC3A |
| SNHG11,DLEU1,FAM87A,AL512652.1,ST7-OT4,ZRANB2-AS2,FTX,DLEU2 | hsa-mir-7-5p | IRS2,NDFIP2 |
| SNHG11,LINC00476,DLEU1,FAM87A,C3orf35,LINC00449,AL512652.1,AL137003.1,LINC00365,RBM26-AS1,DNMBP-AS1,ZRANB2-AS2,MIR210HG,AL928654.1,PAN3-AS1 | hsa-mir-93-5p | NHLRC3,RB1 |
| LINC00476,DLEU1,AL512652.1,ST7-OT4,STK24-AS1,LINC00365,DNMBP-AS1,DLEU2,AL121574.1 | hsa-mir-9-5p | KLF5,SERINC5 |
| DLEU1,FAM87A,ST7-OT4,DLEU2,GAS6-AS1 | hsa-mir-96-5p | EIF4EBP2,NHLRC3 |
| DLEU1,MIR181A2HG,LINC00365,ANKRD10-IT1,FTX,DLEU2,MYCBP2-AS1,CR381653.1 | hsa-mir-98-5p | FNDC3A,IRS2,NEK3,NHLRC3 |

lncRNAs, long non-coding RNAs; miRNAs, microRNAs; mRNAs, messenger RNAs

**Supplementary Table 3.** LncRNAs in the risk model may target specific miRNAs and mRNAs.

| lncRNAs | miRNAs | mRNAs |
| --- | --- | --- |
| SNHG11,FAM87A,HAR1A | hsa-mir-103a-3p | AMOT,YWHAH |
| SNHG11,DNMBP-AS1,MIR210HG | hsa-mir-106a-5p | AKAP11,RB1 |
| SNHG11,FAM87A,HAR1A | hsa-mir-107 | AMOT,CDK8,YWHAH |
| SNHG11,HAR1A,DNMBP-AS1,MIR210HG | hsa-mir-122-5p | SLC7A1 |
| FAM87A | hsa-mir-124-3p | CDCA7,SLC7A1 |
| FAM87A | hsa-mir-1271-5p | EIF4EBP2 |
| DNMBP-AS1 | hsa-mir-1297 | RNF6 |
| SNHG11,MIR210HG | hsa-mir-132-3p | RB1 |
| SNHG11,FAM87A | hsa-mir-141-3p | IPO5,YAP1 |
| FAM87A,MIR210HG | hsa-mir-145-5p | KLF5,SERINC5 |
| SNHG11 | hsa-mir-155-5p | VPS36 |
| FAM87A,MIR210HG | hsa-mir-16-5p | AKAP11,HOXA10,SLC39A10,TM9SF2,TPT1,YWHAH |
| SNHG11,FAM87A,DNMBP-AS1,AL928654.1 | hsa-mir-17-5p | AKAP11,NHLRC3,RB1 |
| MIR210HG | hsa-mir-182-5p | HOXA9 |
| DNMBP-AS1,AL590483.1 | hsa-mir-192-5p | RB1 |
| FAM87A,MIR210HG | hsa-mir-195-5p | AKAP11,HOXA10,TM9SF2,YWHAH |
| SNHG11,FAM87A | hsa-mir-200a-3p | IPO5,YAP1 |
| STK24-AS1,AC019330.1 | hsa-mir-204-5p | FARP1 |
| FAM87A | hsa-mir-205-5p | AMOT |
| FAM87A,DNMBP-AS1,MIR210HG | hsa-mir-206 | FNDC3A |
| SNHG11,MIR210HG | hsa-mir-212-3p | RB1 |
| SNHG11,FAM87A,HAR1A,DNMBP-AS1,MIR210HG | hsa-mir-214-3p | DOCK9,NFIC |
| DNMBP-AS1,AL590483.1 | hsa-mir-215-5p | RB1 |
| DNMBP-AS1 | hsa-mir-217 | DACH1 |
| SNHG11,FAM87A,MIR210HG | hsa-mir-218-5p | DOCK9,MBNL2 |
| SNHG11,FAM87A | hsa-mir-31-5p | MZT1 |
| SNHG11,DNMBP-AS1,MIR210HG | hsa-mir-372-3p | MBNL2 |
| FAM87A,MIR210HG | hsa-mir-424-5p | HOXA10,YWHAH |
| FAM87A,MIR210HG | hsa-mir-497-5p | HOXA10,YWHAH |
| SNHG11,FAM87A,DNMBP-AS1,AL928654.1 | hsa-mir-519d-3p | NHLRC3,RB1 |
| FAM87A,DNMBP-AS1,MIR210HG | hsa-mir-613 | FNDC3A |
| SNHG11,FAM87A | hsa-mir-7-5p | IRS2,NDFIP2 |
| SNHG11,FAM87A,DNMBP-AS1,MIR210HG,AL928654.1 | hsa-mir-93-5p | NHLRC3,RB1 |
| STK24-AS1,DNMBP-AS1 | hsa-mir-9-5p | KLF5,SERINC5 |
| FAM87A | hsa-mir-96-5p | KLF5,SERINC5 |

lncRNAs, long non-coding RNAs; miRNAs, microRNAs; mRNAs, messenger RNAs
